# Exploring obesogenic environments: the design and development of the migrant obesogenic perception of the environment questionnaire (MOPE-Q) using a sample of Iranian migrants in Australia

**DOI:** 10.1186/1471-2458-14-567

**Published:** 2014-06-06

**Authors:** Maryam Delavari, Anders Larrabee Sønderlund, David Mellor, Mohammadreza Mohebbi, Boyd Swinburn

**Affiliations:** 1 WHO Collaborating Centre for Obesity Prevention, Faculty of Health, Deakin University, Melbourne, Australia; 2 School of Psychology, College of Life and Environmental Sciences, the University of Exeter, Exeter, UK; 3 School of Psychology, Faculty of Health, Deakin University, Melbourne, Australia; 4 Biostatistics Unit, Faculty of Health, Deakin University, Melbourne, Australia; 5 School of Population Health, University of Auckland, Auckland, New Zealand

**Keywords:** Environment, Obesity, Migrants, Iranians, Iran, Australia, Questionnaire development, Psychometric evaluation

## Abstract

**Background:**

Although there are a number of studies examining the effect of migration on obesity, these studies tend to focus on the role of acculturation in this relationship. However, there are indications that the change in environment may also be an important factor. Indeed, there is a considerable lack of psychometric tools designed to assess the association between environment and migrant health behaviour. The current study aimed to assess the literature on the link between environment and health for migrants, and on the basis of this information, design and develop the Migrant Obesogenic Perception of the Environment questionnaire (MOPE-Q). The MOPE-Q is the first comprehensive measure of the impact of environmental factors on migrant health behaviour related to physical activity, food habits and body image concern, as well as weight change.

**Methods:**

Using a systematic approach, an initial pool of items for the questionnaire was developed and refined on the basis of rigorous content and face validity assessments and factor analysis. Further, reliability tests and test re-test studies were undertaken. Differences between Iranian and Australian environmental factors as they relate to obesogenic behaviour were explored using the developed measure.

**Results:**

A total of 36 items were developed for the MOPE-Q. Principal factor analysis identified three similar factor structures of environmental factors related to obesity (categorized in terms of facilitators, barriers and pressures) for each country. The final questionnaire consisted of four distinct subscales pertaining specifically to the Australian environment and five subscales pertaining to the Iranian environment, accounting for 59% and 63%, respectively, of the total variance in obesity rates. Data suggests that the MOPE-Q is a reliable and valid self-report measure for assessing the relationship between environmental factors linked to obesity and obesogenic behaviour for this particular migrant group.

**Conclusion:**

The variations in environmental factors linked to obesity behaviour between home (Iran) and host (Australia) countries have been incorporated into the MOPE-Q instrument which has shown good psychometric properties. The MOPE-Q can be adapted and applied to other environments and populations to help explain changes in diet, physical activity patterns and body weight in migrant groups as they acculturate.

## Background

As early as 1998, the World Health Organization (WHO) declared obesity to be a problem of epidemic proportions in most Westernized countries, contributing to a wide range of health and social consequences [1]. Specifically, over the last three decades, obesity has become a major public health concern across high, middle and some low income countries where Western behavioural and dietary patterns are evident [2,3]. Migrant populations represent a particularly interesting case because of the similar, but accelerated, nutritional transition which they experience when moving from low or middle income countries to high-income western countries [4,5]. A growing number of studies in high income countries have demonstrated a stepwise increase in the prevalence of obesity among migrants [5]. In Western societies, migrants are often healthier than local residents at the time of arrival due to the ‘healthy migrant effect’ of the immigration process. That is, people typically have to be in considerably good health to be eligible for immigration. They are therefore generally in better physical health than their hosts. However, typically the health of migrants regresses to the host country’s norm after a some years of residency [6], and a number of studies have reported that the body mass index (BMI) of migrants can quite rapidly increase beyond that of the local population [7,8]. People’s behaviors are heavily influenced by their physical, social and cultural environments. In terms of obesogenic behaviour, the strength of these influences significantly overwhelms other factors, such as those related to genetics [3,9]. As such, socio-ecological models, such as the ANGELO (Analysis Grid for Environments Linked to Obesity) framework [10], describe the physical and socio-cultural, economic and policy dimensions of the community in which they live and which influence their diet and physical activity patterns. Specifically, the ANGELO framework broadly divides obesogenic environments into two levels (micro/settings and macro/sectors) and four types (physical, economic, policy and socio-cultural). This model has been used over the past 15 years for many different purposes relating to obesogenic environments and it has proved to be very robust [11-13]. Thus, the ANGELO is wide in scope, and acknowledges the notion that obesity prevention in migrant populations requires a broad environmental focus on determinants of obesity, beyond the individual level. While there seems to be greater awareness of the need for creating community initiatives aimed at reducing obesity based on comprehensive frameworks such as the ANGELO [12], the evidence base underpinning the knowledge on which particular issues relate to obesity rates in migrant populations is sparse.

As mentioned above, many migrant groups adopt obesogenic behaviours and experience weight gain as they acculturate to their new environment. This experience particularly relates to immigrants from countries with a low or medium human developmental index (HDI) to countries with a high HDI [14]. However, it is not uniform across all migrant groups, and may differ as a result of factors such as country of origin and degree of acculturation [6]. Acculturation relates to the immigrant adoption of the attitudes, values and behavioural patterns of the host culture to which they have migrated. This also includes health behaviours, such as those connected to obesity [4,15]. A large number of studies show that the relationship between obesity and acculturation differs between specific immigrant groups with various populations demonstrating elevated, reduced, or unchanged weight in relation to the degree of acculturation. For example, Fitzgerald and colleagues found an overall positive association between the degree of acculturation and body weight status in Mexican-Americans [16] – a finding which has been supported by other studies [17,18]. Further, while there is limited research investigating gender differences in the association between BMI and acculturation, Lee and colleagues found no significant relationship between acculturation and obesity in their female sub-sample [19]. Other studies have found negative or mixed associations, including less obesity in migrant women [20-22]. This observation reinforces the argument that, in all likelihood, a complex interaction between culture, ethnicity, gender and place of origin impacts on a range of health-related factors, including obesity. That is, the migrant carries personal factors – some fixed like gender, and others which change temporally such as age – across countries which interact with the change in environments between countries. Broad-scale approaches to obesity prevention in migrant populations are thus more likely to succeed if environmental obesogenic factors are understood and used to shape relevant initiatives and interventions. This interaction is what is hypothesized to help explain the variations in changes in diet, physical activity patterns and weight among migrants [23].

In order to develop the evidence base for explaining predictors of unhealthy weight gain post-migration, phenomena such as the ‘healthy migrant effect’, the ANGELO framework and acculturation theory need to be integrated and synthesized. Thus, assumptions about the relative importance of each of the constructs, in terms of obesity-linked behaviour, need to be tested. While most studies have demonstrated that the foreign-born members of the community experience some changes in their BMI post migration, the majority of these studies fail to measure environmental elements that may directly or indirectly affect migrants’ obesogenic behaviour [6]. There are several reasons for this. First, comparison groups often comprise natives of the host country rather than natives living in the country of origin. That is, second- and third-generation immigrants may be used as controls. Second, few studies of international migration contain information about the migrants’ past way of life and social environment, with most focusing solely on migrants’ degree of acculturation. Third, most existing research about acculturation has relied on surrogate measures of acculturation (e.g. place of birth, language, length of residency in the host country) rather than standardized scales of acculturation. Fourth, previous studies show migrants are generally healthier than their local counterparts and attribute this result to the positive selection of migrants on health. Most of these studies, however, do not examine the extent to which migrants’ health varies as a function of pre-migration health environment or their specific reasons for migration (e.g. refugee, family, work force, educational opportunity, etc.) [6,23].

Thus, while acculturation theories may be useful for predicting migrants’ post-immigration obesity rates, they are largely based on individual migrant characteristics and obesity status, with little understanding of the effects of changing environments on migrant BMI. In light of this, similarities and differences between health-related pre- and post-migration environmental elements should also be investigated to help both improve our understanding of, and inform public health efforts to prevent, obesity. This is especially critical for increasingly multicultural countries like Australia. Thus, systematic research on this topic is required. To do this effectively and empirically, a valid and reliable measure that can distinguish the key obesogenic elements in specific environments, is needed. In previous study, we sought to understand the changes in environment experienced by migrants to Australia and how this may have impacted on their health [23]. Results indicated an emphasis on both individual and environmental factors in this context. In light of this, the current paper describes the design and methodological components of the Migrant Obesogenic Perception of the Environment questionnaire (MOPE-Q). The developed scale also provides a snapshot of how factors in home and host countries have been experienced differently by a sample of Iranian migrants in Australia.

## Method

In developing the questionnaire, we conducted a structured approach including, 1) transitional validity tests (i.e. content validity, face validity, and factor extraction), and 2) reliability tests (i.e. equivalence reliability to check the internal consistency of the scale and finally stability reliability, test re-test) [24].

### Procedure

To test the validity, reliability and overall accuracy of the developed questionnaire, trained research assistants administered the questionnaire to volunteer participants. Data collection occurred between 19 August and 10 September 2012. The questionnaire took approximately 45 minutes to complete. Any difficulties experienced by participants in understanding the questions were dealt with by bi-lingual (Farsi and English) research assistants hired and trained for the research project. Informed consent was obtained from participants, all of whom volunteered after reading the plain language statement describing the study. At the end of the survey administration, each participant received a movie ticket voucher as a token of appreciation.

### Participants

Guidelines for the sample size required for factor analysis relate to the number of items included in an instrument. Some authors suggest that a ratio of five to ten participants for each item is required [25]. However, others suggest a minimum sample size (e.g. 100 to 150 without missing data) should be sufficient [26]. Thus, for this research, a total sample of 150 Iranian adults was sought for conducting exploratory factor analysis (EFA). We recruited participants through Iranian community associations, namely the Iranian Students of Victoria Alumni (ISVA), the Iranian Society of Victoria (ISOV), and the Iranian Language Weekend School in Melbourne. The initial research sample included an equal number of male and female adults. The research was approved by the Deakin University Ethics Committee (HEAG-H 90_2011). Inclusion criteria were: (i) born in Iran; (ii) being between the ages of 18 and 65 years; (iii) being in Australia for more than 5 months and less than 30 years (we chose this time frame to ensure that participants could accurately recollect their lifestyle in Iran post the 1979 revolution). Initially, 200 individuals were recruited into the study over two separate occasions. However, as 90 people did not either attend the scheduled group or complete the study, another 50 individuals were recruited to reach the required sample size so that the total number of participants was 152. Males comprised 55.3% (*n* = 84) of the total sample, and females 44.7% (*n* = 68). The age-range was 19-65 with a mean age of 34 years old. The time that had passed since immigration to Australia was between six months and 28 years, with the average being approximately four years.

### Transitional validity

If the proposed MOPE-Q is to achieve its aims, the measure should be able to distinguish between environmental factors potentially impacting on participants’ food habits, physical activity and body image concern in different locations. To do this we employed content and face validity analysis and factor extraction.

To assure transitional validity, items were generated from a number of resources, including a review of the literature, a previously conducted qualitative study on Iranian migrants in Victoria [23], the ANGELO framework [10], as well as considerable pilot work to refine wording and content. Each subscale is represented by multiple variables/items likely to be influenced by each of the suggested domains. The initial item pool consisted of 44 items comprising eight sub-scales (Table 1). Each item measured an interaction between either an obesogenic (barrier for healthy behaviour) or a healthy (facilitator for healthy behaviour) environmental factor and individual's obesity behaviour (see Table 1). A 7-point Likert scale ranging from 1 = healthy, 'strongly disagree' to 7 = obesogenic, 'strongly agree' was used for each item. Each item was completed twice: once for the home environment (Iran) and once for the host environment (Australia). Thus, the questionnaire included domains which arose from the literature (specifically in relation to the experiences of immigrants but also the literature on the environmental influences on diet and PA more broadly) and the synthesis of previously-reported FGDs with Iranian immigrants. In addition, the ANGELO framework was used as an overarching framework to ensure that potentially important domains were included in the initial questionnaire.

**Table 1:** Content validity and face validity of MOPE-Q

**Original items**	**Comments final decision**	**Amended items**	**Obesogenic Likert scale**
**Physical environment for physical activity (PEPA)**			
1. Air pollution inhibited your ability to engage in daily physical activity in Iran	Keep and revise	1. Air pollution discourages me from being physically active	7
2. The availability of "green space" such as parks encouraged you to engage in physical activity in Iran	Keep and revise	2. The accessibility of "green spaces" such as parks encourages me to be physically active	1
3. Accessibility to foot paths and cycle paths encouraged you to be more physically active in Iran	Keep and revise	3. Accessibility to walking and cycling paths encourages me to be physically active	1
4. Street safety encouraged you to be more physically active in Iran	Keep and revise	4. Lack of safety on the streets (e.g. Harassment or traffic) discourages me from being physically active	7
5. The availability of quality hobby spaces encouraged you to participation in active leisure activities in Iran	Keep and revise	5. The availability of quality leisure facilities (e.g. gyms and pools) encourages me to be physically active	1
**Physical environment for food habits (PEFH)**			
6. You had easy access to a variety of food service outlets which served unhealthy food in Iran	Keep and revise split into two groups(unhealthy food/service outlet)	6. Ready access to unhealthy food service outlets encourages me to eat unhealthy foods	7
-	Added	7. Ready access to unhealthy foods such as packaged snacks, soft drinks and fatty meats encourages me to eat unhealthy foods	7
-	Need a new question because it is not the flip side of the unhealthy food outlets	8. Ready access to healthy food service outlets encourages me to eat healthy foods	1
7. You had easy access to a variety of healthy food such as fruits, vegetables, whole grains and fish in Iran	Keep and revise, split into two healthy food groups	9. Ready access to fruit, vegetables and whole grains encourages me to eat healthy foods	1
-	Added	10. Ready access to lean meat and fish encourages me to eat healthy foods	1
**Public policy for physical activity (PPPA)**			
8. Expertise and information could help you to have regular physical activity was available/easily accessed in Iran	Keep and revise	11. Ready access to reliable expertise and information encourages me to be physically active	1
9. Regular usage of exercise facilities (e.g., gym and pool) was expensive in Iran	Drop, Expensive in both countries	-	7
10. Work demands (i.e. typically long working hours) made having a healthy life style difficult in Iran	Keep and revise	12. Work demands (i.e. working long hours) discourages me from being physically active	7
11. Regulations surrounding appropriate dressing/public behaviour inhibited you from doing regular PA in Iran	Keep and revise	13. Government rules about appropriate clothes and behaviours discourage me from being physically active in public	7
**Public policy for food habits (PPFH)**			
12. Nutritional expertise and information helped you to have a healthy diet was available/easily accessed in Iran	Keep and revise	14. Ready access to reliable expertise and information encourages me to have a healthy diet	1
13. Consumption of healthy food was expensive in Iran	Keep and revise, split into two groups	15. Fruit and vegetables are expensive and this discourages me from having a healthy diet	7
-			16. Lean meat and fish are expensive and this discourages me from having a healthy diet	7
14. An unhealthy diet was cheaper than a healthy diet in Iran	Keep and revise	17. Unhealthy food is cheap and this encourages me to have an unhealthy diet	7	
**Public policy for body concern (PPBC)**				
15. Dress codes (regulations surrounding appropriate dressing) influenced your eating behaviour in Iran	Keep and revise, question was around eating but the sense should be around body size	18. Government rules about appropriate clothes encourages a lack of concern about my body weight and size	7	
**Socio-cultural environment for physical activity (SCPA)**				
16. Family, friends and community gathering promote sedentary lifestyles in Iran	Keep and revise, Possible confusion with food - only need one question of PA or sedentary behaviour	19. Social gatherings of families, friends and communities inhibit me from being physically active	7	
17. Family, friends and community gatherings promote active lifestyles in Iran	Drop	-	1	
18. Regular/daily exercise was a part of the culture in Iran	Keep and revise	20. There is a culture of being physically active and this encourages me to be physically active	1	
19. You preferred active transport over motorized transport (cars, lifts, escalators) in Iran	Keep and revise	21. The cultural norm of using your own car discourages me from walking, cycling or taking public transport	7	
20. Religious leaders had a strong influence on your beliefs/behaviour related to diet and activity in Iran	Drop, the question was unclear, whether their influence promotes or inhibits healthy lifestyle. Also, asking about religious leaders’ influence could be a sensitive issue for this specific migrant group	-	Unclear	
21. Celebrities and fashion models had a strong influence on your beliefs/behaviour related to diet and activity in Iran	Drop, too general and unclear whether their influence promotes or inhibits healthy lifestyle	-	Unclear	
22. Seeing of people doing exercise in the public motivated you to be more physically active in Iran	Keep and revise	22. Seeing people doing physical activity in public encourages me to be physically active	1	
23. The media (e.g. TV programs, magazines) encouraged you to being more physically active in Iran	Keep and revise	23. The media (e.g. TV programs, magazines) encourages me to be physically active	1	
**Socio-cultural environment for food (SCFH)**				
24. Family, friends and community gathering leaded you to consume a lot of fatty and sugary food in Iran	Keep and revise, Using 'fatty and sugary' rather than 'unhealthy' foods	24. The cultural norms at social gatherings of families, friends and communities encourages me to eat a lot of fatty and sugary foods	7	
25. The media encouraged you to follow a healthy diet in Iran	Keep and revise	25. The media encourages me to follow a healthy diet	1	
26. You were exposed to unhealthy food (i.e. high in fat and sugar) through media advertising in Iran	Keep and revise	26. The high level of advertising encourages me to eat unhealthy foods	7	
**Socio-cultural environment for body size concern (SCBC)**				
27. People had negative attitudes/beliefs about overweight/obesity in Iran	Keep and revise	27. People’s negative attitudes or beliefs about overweight or obese people encourages me to be concerned about my body size and weight	1	
28. Good looking and beautiful for girls meant being thin in Iran	Drop, as specifically asking from only one gender, male or female, merged to items about marriage with considering good looking	-	1	
29. Good looking/beautiful for girls meant having a muscular body in Iran	Drop, as above	-	1	
30. Good looking/beautiful for girls meant having some extra weight in Iran	Drop, as above	-	7	
31. Good looking/handsome for boys meant being thin in Iran	Drop, as above	-	1	
32. Good looking/handsome for boys meant having a muscular body in Iran	Drop, as above	-	1	
33. Good looking/handsome for boys meant having some extra weight (being chubby) in Iran	Drop, as above	-	7	
34. You would prefer to marry a slender (slim) girl/boy in Iran	Keep and revise	28. If I was considering marriage, I would feel a lot of pressure to marry a slim person	1	
35. You would prefer marry to a girl/boy with a muscular body in Iran	Keep and revise	29. If I was considering marriage, I would feel a lot of pressure to marry a muscular person	1	
36. You would prefer marry to a girl/boy with some extra weight (being chubby) in Iran	Drop, unclear	-	7	
37. When trying to lose weight, you were more likely choose unhealthy strategies rather than healthy lifestyle in Iran	Keep and revise	30. If I want to lose weight, I would feel a lot of pressure to choose unhealthy strategies such as rigorous dieting, diet pills or liposuction rather than regular physical activity and healthy diet	1	
38. Observing people's body shapes acts as a wakeup call for not wanting to get obese in Iran	Keep and revise	31. Seeing overweight or obese people in public makes me worry about becoming obese myself	1	
39. You felt too much pressure to be slim from your partner and family in Iran	Keep and revise	32. I feel a lot of social pressure to be slim	1	
40. You felt too much pressure to be slim from your Peers/friends in Iran	Keep and revise	33. I feel a lot of social pressure to be fit and muscular	1	
41. You felt too much pressure to be slim from Media in Iran	Keep and revise	34. I feel a lot of pressure from the media to be slim	1	
42. People critically evaluated your body image in Iran	Keep and revise	35. People critically evaluate my body size	1	
43. Seeing of people being conscious about their body size leads an individual to watch his/her own weight in Iran	Keep and revise	36. Seeing people being conscious about their body size leads me to watch my weight and size	1	

After the survey design was completed, face validity of the questionnaire was tested through the use of Lynn’s criteria [27]. The survey was piloted with six Iranian university students resident in Australia and then six health professionals, functioning as an expert panel. During face-to-face interviews, participants indicated whether each of the 44 items was clear or unclear. Items perceived as confusing or unclear were eliminated or reworded to add clarity to the item pool. Finally, the expert panel came together to produce a final set of items. All items were re-evaluated and then refined or discarded if they were not sufficiently supported by the relevant theories and/or the pilot testing.

Finally, factor analysis was conducted to explore the relationships among the MOPE-Q items and to determine how items were grouped together for each context (i.e. Iranian environment and Australian environment). EFA is used in situations where there is insufficient prior theory and empirical models to represent the structure of association among the measured variables [25,28-30]. EFA, therefore, was an appropriate form of analysis for our research objective. EFA using maximum likelihood estimation was conducted. Specifically, we used principal component analysis followed by varimax rotation [24,28,31] as the EFA approach. Factor loadings should be at least .30, explaining about 10% of the variables within a domain [28,32]. We chose loadings equal to or greater than .40 as the minimal level of interest, or if an item loaded above .40 on multiple factors, it was assigned to the factor with the highest loading [33,34].

### Reliability tests

Equivalence reliability statistics for the questionnaire were evaluated with a series of Cronbach alpha coefficients for each subscale to confirm the internal consistency of the subscales [35,36]. A Cronbach alpha of ≥ .70 for each subscale and for the overall tool is considered acceptable for new scales and indicates that the items in the tool are grouped together [27,36].

Next, test-retest reliability of the MOPE-Q was assessed for the Iran and Australia versions to asses the stability of the final scale for both contexts. To this end, in addition to the sample described above, a second sample of Iranian university students (N = 146) was randomly drawn from the population registry of the Iranian Students of Victoria (ISOV) by the registry office. All students aged between 18 and 50 who had been in Australia for more than 5 months and less than 10 years, (we chose this time frame to ensure that participants could accurately recollect their lifestyle in Iran and the changes that followed), received an invitation letter to participate in this study via email. The survey was emailed to volunteers twice – first, immediately after signing up to participate, and then for the re-test two weeks later. A two-week time interval between the original test and retest is the generally accepted time interval between test and retest [27]. Analyses of the test re-test were undertaken. The bivariate correlation coefficient significant at the .01 level (2-tailed) between the two sets of responses was used in order to produce a preliminary indication of whether consistency was maintained [37].

## Results

### Questionnaire face validity

Table 1 presents the initial item pool, the refinement of the initial questionnaire as well as the final 36 items comprising 8 sub-scales:

1) The physical environment for physical activity (PEPA) (5 items),

2) The physical environment for food habits (PEFH) (5 items),

3) The public policy for physical activity (PPPA) (3 items),

4) The public policy for food habits (PPFH) (4 items),

5) The public policy for body size concern (PPBC) (1 items),

6) The socio-cultural environment for physical activity (SCPA) (5 items),

7) The socio-cultural environment for food habits (SCFH) (3 items),

8) The socio-cultural environment for body size concern (SCBC) (8 items).

### Questionnaire factor extraction

Factor analysis: Of the total 36 items, 25 had factor loadings higher than .40 (across both environments, see Tables 2 and 3). Of these, eight items related solely to the Iranian environment, and another eight items related solely to the Australia environment and nine items were relevant for both environments.

**Table 2:** Factor analysis results for the MOPE-Q – Iranian version

**Items**	**Healthy food environment**	**Social pressure to be fit**	**Physically inactive environment**	**Silent media**	**Unhealthy food environment**
Ready access to fruit, vegetables and whole grains encouraged me to eat healthy foods	.82			.11	
Ready access to lean meat and fish encouraged me to eat healthy foods	.78	.16	.18		
Ready access to healthy food service outlets encouraged me to eat healthy foods	.75	.18	.13	.18	
Ready access to reliable expertise and information encouraged me to have a healthy diet	.46	.17	.13	.18	
People critically evaluated my body size		.77			
I felt a lot of social pressure to be fit		.73	-.16		
I felt a lot of social pressure to be slim	.25	.71	.14		
Seeing people being conscious about their body size leaded me to watch my weight and size	.23	.69		.23	
Seeing people not doing physical activity in public discouraged me from being physically active		.17	.80	.15	
Lack of accessible "green spaces" such as parks discouraged me from being physically active	.19	-.11	.77	.14	
Lack of accessibility to walking and cycling paths discouraged me from being physically active	.11	-.16	.58	.22	.22
I did not feel a lot of pressure from the media to be slim		.21	.17	.75	
The media did not encourage me to follow a healthy diet	.25		.13	.75	.13
The media (e.g. TV programs, magazines) did not encourage me to be physically active	.17		.37	.68	
Ready access to unhealthy food encouraged me to eat unhealthy foods	.10			.20	.80
Ready access to unhealthy food service outlets encouraged me to eat unhealthy foods		.13	.14	-.27	.77
Unhealthy food was cheap and this encouraged me to have an unhealthy diet	-.22		.11	.14	.74

**Table 3:** Factor analysis results for the MOPE-Q – Australian version

**Items**	**Physically active environment**	**Social pressure to be fit**	**Government liveability policies**	**Unhealthy food environment**
There is a culture of being physically active and this encourages me to be physically active	.67	.22		
The availability of quality leisure facilities (e.g. gyms and pools) encourages me to be physically active	.73			
Accessibility to walking and cycling paths encourages me to be physically active	.77			.13
Seeing people doing physical activity in public encourages me to be physically active	.77	.10	-.12	
The accessibility of "green spaces" such as parks encourages me to be physically active	.71	.16		
I feel a lot of social pressure to be fit	.39	.54	.25	
I feel a lot of social pressure to be slim	.23	.75	-.11	
Seeing people being conscious about their body size leads me to watch my weight and size	.26	.71	.17	-.15
People’s negative attitudes about obese people encourages me to be concerned about my weigh		.73	-.11	.15
people critically evaluate my body size	-.12	.79	.11	
Government rules about clothes and behaviours encourages me to be physically active in public	.19	.11	.60	
Lack of air pollution encourages me to be physically active	-.11		.75	
Street safety (e.g. lack of harassment or traffic) encourages me to be physically active	-.20	.10	.76	-.14
Government rules about cloths encourages me to be concern about my body size			.78	
The high level of advertising encourages me to eat unhealthy foods				.76
Unhealthy food is cheap and this encourages me to have an unhealthy diet	.22		.23	.81
Ready access to unhealthy food service outlets encourages me to eat unhealthy foods				.84

Exploring the results for each country, four subscales were identified for Australia, together accounting for 59% of the total variance. We labeled the extracted subscales based on our understanding and interpretation of the concepts. The subscales related to, 1) *physical active environment* (factor loading: .67-.77), 2) *social pressure to be slim or fit* (factor loading: .54-.79), 3) *government liveability policies* (factor loading: .60-.78), and 4) *unhealthy food environment* (factor loading: .76-.84) (see Tables 2 and 3). The first component measured how the availability of green spaces, walking and cycling paths as well as the culture of physical activity in Australia may encourage Iranian migrants to be physically active. The second subscale, *social pressure to be slim or fit* consisted of five items which measured how the routine social-cultural pressure of fitness, being conscious about body size and people’s negative attitudes about overweight or obese people might pressure Iranian migrants to be concerned about their weight and body size following migration to Australia. The third subscale, *government liveability policies* included four items pertaining to how the Australian government’s ‘liveability’ policies may encourage Iranian migrants to be physically active and concerned about their weight. For example, in Iran there are strict dress codes for males and females, most of which (in particular for females) are not practical for physical activity. Finally, the fourth subscale, *unhealthy food environment* related to the obesogenic food environment in Australia (i.e. ready access to unhealthy food service outlets, cheap unhealthy food and the high level of advertising for unhealthy foods) that could encourage Iranian migrants to have unhealthy food habits.

Five subscales were elicited for life in Iran, accounting for 63% of the total variance. The subscales were respectively labeled as; 1) *healthy food environment*, 2) *social pressure to be slim or fit*, 3) *physically inactive environment*, 4) *silent media,* and 5) *unhealthy food environment*. The first subscale consisted of four items (factor loading: .46-.82). This component related to how ready access to healthy food (i.e. lean meat and fish as well as fruit, vegetables and whole grains), healthy food service outlets in addition to reliable expertise and information could help Iranians in Iran to have a healthy diet. The second subscale, *social pressure to be slim or fit* also included four items (factor loading: .69-.77). This subscale related to the social pressure of being physically fit and conscious of body size, and the extent to which people were critical and/or evaluative of others’ body size. The third subscale, *physically inactive environment* consisted of three items that loaded highly (factor loading: .58-.80) together and relates to the lack of available green spaces, walking and cycling paths as well as the culture of sedentary life in Iran. Three factors for media environment (factor loading: .63-.75) comprised the fourth subscale for Iran, *silent media*. This subscale related to the lack of support through the media (*silent media*) for healthy eating and physical activity. Finally three items (factor loading: .74-.80) comprised the fifth component for Iran, *unhealthy food environment*. This component reflected the existence of environmental factors contributing to unhealthy food habits in Iran. The items related to unhealthy food and also unhealthy food service outlets. At this stage, the proposed MOPE-Q comprised 17 items for each country.

### Questionnaire reliability

#### Equivalence reliability

All Cronbach alpha values were above .70 for the Australian version and also for the Iranian version with the exception of *unhealthy food environment* for Iran (.68). Tables 4 and 5 show the internal reliability coefficients for each subscale and also for the total MOPE-Q scale of each country, Iranian MOPE-Q version and Australian MOPE-Q version.

**Table 4:** Reliability of the MOPE-Q – Iranian version (Cronbach’s alpha values)

**Subscales**	**Cronbach’s alpha (*n*** **= 152)**	**Test-restest (*n*** **= 41)**
Healthy food environment	.76	.71
Social pressure to be fit	.72	.80
Physically inactive environment	.67	.53
Silent media	.70	.64
Unhealthy food environment	.68	.49
All items (scale)	.76	

All correlations significant at p < .01.

**Table 5:** Reliability of the MOPE-Q – Australian version

**Subscales**	**Cronbach’s alpha (*n*** **= 152)**	**Test-restest (*n*** **= 41)**
Physical active environment	.80	.69
Social pressure to be fit	.78	.81
Government liveability policies	.70	.69
Unhealthy food environment	.74	.52
All items (scale)	.73	

All correlations significant at p < .01.

#### Test re-test

The outcome of the test re-test study for Iran and Australia questionnaires is shown in Tables 4 and 5. For Australia, the results revealed significant correlations between time one and time two for each subscales (*r* = .52 to.81, *p* < .01), suggesting strong relationship between participants’ first time and second time responses. For the Iranian questionnaire, the results also revealed significant correlations between time 1 and time 2 for each subscales (*r* = .49 to .80, *p* < .01), suggesting a strong relationship between participants’ first time and second time responses. Of note, the stability of responses was quite strong for the presence of social pressure of fitness in Iran (*r* = .80) which was also the case for Australia (*r* = .81). Compared to other subscales participants were less consistent over the test-retest period in reporting Silent Media in Iran (*r* = .49, p < .01).

## Discussion

Immigrants experience a range of environmental and social changes as they move between home and host environments. These differences in environments often have the potential to impact directly on the immigrant’s physical health, including for example body weight. This study developed and tested a new scale for measuring the impact on immigrants to Australia of the change in environmental determinants of obesity experienced as they migrate from their homeland (Iran) to their host (Australia) country. Employing both qualitative and quantitative processes was relevant in choosing, reviewing, and extracting items. This was particularly important when attempting to employ items across two different settings that have been experienced by a sample of Iranian immigrants in Victoria, Australia. Furthermore, using qualitative information from previous study [8] on Iranian migrants enabled us to determine different aspects of Iranian migrants’ experiences rather than solely relying on scholars’ theories and guidance from previous obesity and migrants’ health research.

The emphasis on physical activity environments and government liveability policies in Australia as important influences on healthy weight behaviour in Australia was notable. By contrast Iran was perceived by most as an environment promoting physical inactivity. In terms of food environment, the majority of our participants also indicated that the existence of a generally unhealthy food environment in Australia discouraged them from following a healthy diet. The food environment was also a factor in Iran – however, for Iran there were relatively high factor loadings for a healthy food environment. Together, these results underscore the overall importance of food environment and social norms for healthy weight behaviour in both Australia and Iran.

Another factor mentioned frequently by participants related to the media as a silent environmental key factor in Iran and suggested that if the media was positive in terms of health promotion then it could possibly encourage Iranians to have healthier weight-related behaviours. This is particularly interesting as past research has not examined the effect of Iranian media on Iranians body image concern [38]. Our results, however, do not warrant clear conclusions, but encourage future research to investigate this relationship further.

Overall, adding the environmental dimension in assessment of the relationship between acculturation and migrant obesity has undoubtedly increased the complexity of the migration-obesity paradigm. Ultimately, however, it can be argued that considering both culture and environment in this context affords a truer reflection of migrant experiences, and thus a better understanding of the impact of migration on obesity.

## Conclusion

The influences of obesogenic environments on behaviours are inherently complex and most models have considered the relationship at one point in time. This study takes account of the changing environments as migrants move from one country to another, and experience changes in the obesogenicity of the different aspects of the environment (including socio-cultural environments), that they need to meld with their own culture of beliefs, attitudes, cuisine and so on. This study has started to construct this dynamic aspect of behavioural-environment interaction to build on understandings of cross-sectional relationships. Thus, the MOPE-Q is the first questionnaire developed to measure perceived environmental changes in the determinants of obesity in migrant populations. The factor analysis provided valuable information in addition to reliability testing and confirmed the MOPE-Q as a valid and reliable instrument to be used among Iranian migrant groups in Australia and could be further tested on Iranian migrants in other countries with the same socio-cultural, economic and policy status. The outcome of rigorous validity and reliability tests for both the Iran questionnaire and the Australia questionnaire revealed that responses were very consistent over time, and thus provided testament to the soundness of the instruments’ psychometric properties.

In future research, this questionnaire may also help develop evidence-based and culturally appropriate obesity prevention in Iran and other similar countries.

Further, insight into obesogenic socio-cultural factors in Australia may also be gained through use of the MOPE-Q. It may have implications for obesogenic factors which are changeable in Australia as well as other societies.

In terms of future research, we will use the MOPE-Q for further study of the effect of environmental determinants of obesity on food habits, physical activity as well as the BMI of Iranian migrants in Victoria, Australia. It is also possible that the instrument could be applied to other ethnic groups, although researchers would need to take into account the cultural relevance, migration experiences, home and host environments, and the perceived diversity of obesogenic factors for each targeted migrant group. Finally, we believe that the MOPE-Q could be modified for application in other high income countries that mirror the socio-cultural and economic environment in Australia.

### Ethical approval

The Deakin University Ethics Committee granted ethical approval for this study (HEAG-H 90_2011).

## Competing interests

The authors declare that they have no competing interests.

## Authors’ contributions

All authors have made substantive intellectual contributions to this study,1) MD, DM, MM and BS have made substantial contributions to conception and design,2) MD carried out acquisition of data,3) MD, MM, BS and DM performed analysis and helped to interpret the data,4) MD and ALS has been involved in drafting the original manuscript,5) MD, BS, ALS and DM have been involved in revising the manuscript critically for important intellectual content,6) All authors read and approved the final manuscript to be published.

## Authors’ information

Dr. Maryam Delavari is a PhD Scholar in WHO Collaborating Centre for Obesity.

Prevention, School of Health& Social development, Deakin University, Australia.

Anders L. Sønderlund is a PhD researcher and research fellow at the School of Psychology, College of Life and Environmental Sciences, University of Exeter, UK.

Dr. Mohammadreza Mohebbi is a senior research fellow in Biostatistics Unit, Faculty of Health, Deakin University, Melbourne, Australia.

David Mellor is a Professor in the School of Psychology and Director of the professional postgraduate programs, Deakin University, Australia.

Boyd Swinburn is a Professor of Population Nutrition and Global Health, University of Auckland as well as Alfred Deakin Professor and Director of WHO Collaborating Centre for Obesity Prevention, Deakin University, Australia.

## Pre-publication history

The pre-publication history for this paper can be accessed here:

http://www.biomedcentral.com/1471-2458/14/567/prepub
